# Structural Characterization of New Microcystins Containing Tryptophan and Oxidized Tryptophan Residues 

**DOI:** 10.3390/md11083025

**Published:** 2013-08-21

**Authors:** Jonathan Puddick, Michèle R. Prinsep, Susanna A. Wood, Christopher O. Miles, Frode Rise, Stephen Craig Cary, David P. Hamilton, Alistair L. Wilkins

**Affiliations:** 1 Cawthron Institute, Private Bag 2, Nelson 7042, New Zealand; E-Mails: jonathan.puddick@cawthron.org.nz (J.P.); susie.wood@cawthron.org.nz (S.A.W.); 2 Chemistry Department, University of Waikato, Private Bag 3105, Hamilton 3240, New Zealand; E-Mail: a.wilkins@waikato.ac.nz; 3 Department of Biological Sciences, University of Waikato, Private Bag 3105, Hamilton 3240, New Zealand; E-Mails: c.cary@waikato.ac.nz (S.C.C.); d.hamilton@waikato.ac.nz (D.P.H.); 4 Norwegian Veterinary Institute, P.O. Box 750 Sentrum, N-0106 Oslo, Norway; E-Mail: chris.miles@vetinst.no; 5 Department of Chemistry, University of Oslo, N-0315 Oslo, Norway; E-Mail: frode.rise@kjemi.uio.no

**Keywords:** microcystin, tryptophan oxidation, *N*‑formylkynurenine, mass spectrometry, nuclear magnetic resonance spectroscopy, *Microcystis* CAWBG11

## Abstract

Microcystins are cyclic peptides produced by cyanobacteria, which can be harmful to humans and animals when ingested. Eight of the (more than) 90 microcystin variants presently characterized, contain the amino acid tryptophan. The well-researched oxidation products of tryptophan; kynurenine, oxindolylalanine, and *N*-formylkynurenine, have been previously identified in intact polypeptides but microcystin congeners containing oxidized tryptophan moieties have not been reported. Liquid chromatography-tandem mass spectrometric analysis of an extract of *Microcystis* CAWBG11 led to the tentative identification of two new tryptophan-containing microcystins (MC‑WAba and MC-WL), as well as eight other microcystin analogs containing kynurenine, oxindolylalanine and *N*‑formylkynurenine (Nfk). Investigation of one of these congeners (MC‑NfkA) by nuclear magnetic resonance spectroscopy was used to verify the presence of Nfk in the microcystin. Liquid chromatography-mass spectrometry analysis of a tryptophan oxidation experiment demonstrated that tryptophan-containing microcystins could be converted into oxidized tryptophan analogs and that low levels of oxidized tryptophan congeners were present intracellularly in CAWBG11. MC-NfkR and MC-LNfk were detected in standards of MC-WR and MC-LW, indicating that care during storage of tryptophan-containing microcystins is required.

## 1. Introduction

Microcystins (MCs) are cyclic heptapeptides produced by cyanobacteria which can be harmful to humans and animals upon ingestion. They range in toxicity from non-toxic to highly toxic (50 µg/kg) according to their ability to inhibit the important eukaryotic regulatory enzymes, serine/threonine protein phosphatases 1 and 2A [1]. They are generally comprised of the unique β-amino acid, 3*S*-amino-9*S*-methoxy-2*S*,6,8*S*-trimethyl-10-phenyldeca-4,6-dienoic acid (Adda), d-glutamic acid (Glu), d-alanine (Ala), *N*-methyldehydroalanine (Mdha), d-*erythro*-β-methylaspartic acid (Masp), and two variable l-amino acids [2]. To date, at least 90 different microcystin congeners have been characterized [3], mostly due to substitutions of the variable l-amino acids in positions two and four, although modifications have been reported for all of the amino acids [4]. 

**Scheme 1:**
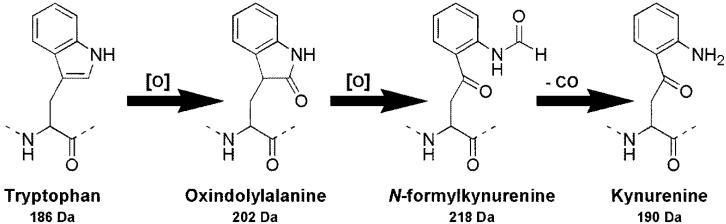
Products from the oxidation of tryptophan in proteins according to Taylor *et al*. [8].

Oxidation of the amino acid, tryptophan (Trp), was first reported in 1903 [5] and by 1931, kynurenine (Kyn) was identified as a by-product of the biological oxidation of tryptophan [6]. Since then, the pathway for the enzymatic degradation of tryptophan has been expanded to include four intermediates which result in either kynurenic acid or quinolinic acid [7]. Whilst the function of this oxidative pathway is to degrade tryptophan residues, the oxidation of tryptophan in intact polypeptides is also apparent [8,9,10,11]. The major products of this oxidation are oxindolylalanine (Oia), *N*‑formylkynurenine (Nfk) and Kyn (Scheme 1), with Nfk being the most abundant [8,9]. 

It is still unclear whether the oxidation of tryptophan residues in polypeptides is due to natural levels of reactive oxygen species in the cell [12], cellular oxidative stress [13], post-translational modification of the tryptophan [8], or is an artifact of sample handling. It has been shown that the production of tryptophan oxidation products is promoted by increased levels of reactive oxygen species [9,14], therefore oxidation of tryptophan may be an artifact of sample handling, although, this does not exclude oxidized tryptophan occurring naturally inside cells. Whilst eight tryptophan-containing microcystins have been reported previously [15,16,17,18,19,20,21], the presence of oxidized tryptophan residues in microcystins has not. 

We recently reported a new tryptophan-containing microcystin congener from *Microcystis* CAWBG11, MC-WA [16]. The presence of a microcystin with a structure similar to that of MC-WA, but with 32 Da additional mass, was also noted (MC-1014). During the present study, two further tryptophan-containing microcystins from *Microcystis* CAWBG11 were characterized by liquid chromatrography-tandem mass spectrometry (LC-MS/MS). Further analysis of the previously noted MC-1014 by nuclear magnetic resonance (NMR) spectroscopy revealed it to be a new microcystin containing the tryptophan oxidation product, Nfk. This supports the tentative identification by LC-MS/MS of seven further microcystin analogs containing the oxidized tryptophan residues Nfk, Oia, and Kyn. 

## 2. Results and Discussion

### 2.1. Liquid Chromatography-Tandem Mass Spectrometric Identification New Microcystins Containing Tryptophan and Oxidized Tryptophan Residues

**Figure 1:**
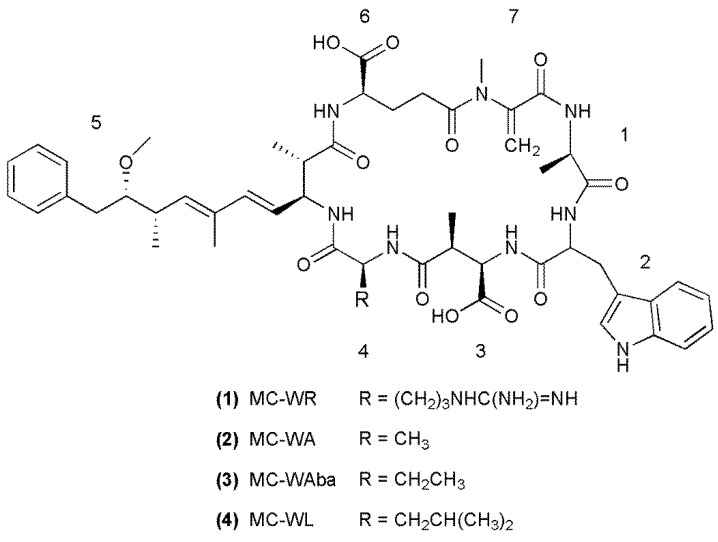
Structures of the tryptophan-containing microcystin congeners found in *Microcystis* CAWBG11, with amino acid numbering (1–7) indicated (Configuration for **3** and **4** is assumed).

A methanol extract of *Microcystis* CAWBG11 was analyzed in-depth by LC-MS/MS. This led to the identification of a multitude of conventional microcystin congeners [21], including the known [15] MC-WR (**1**), the recently reported [16] MC-WA (**2**), and two new analogs containing tryptophan residues, MC-WAba (**3**) and MC-WL (**4**, Figure 1).

**Figure 2:**
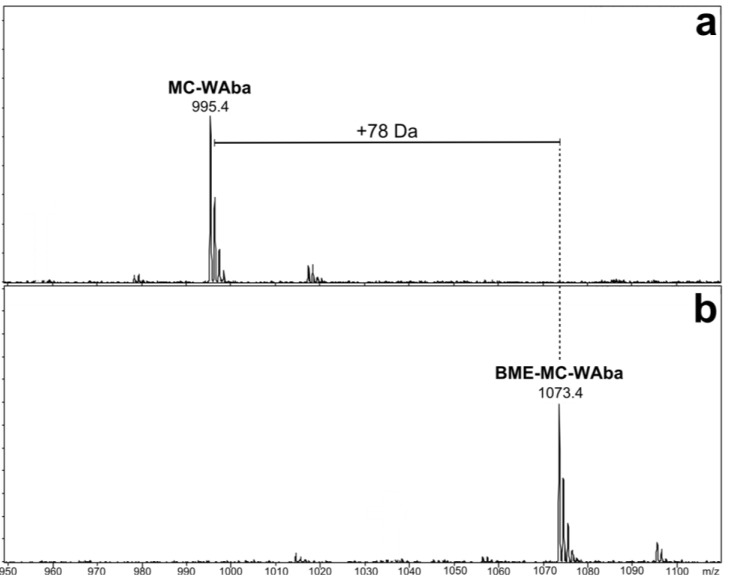
Negative ion liquid chromatography-mass spectra of MC-WAba (**a**) prior to β-mercaptoethanol (BME) derivatization and (**b**) after two hours of incubation at 30°C.

The MS/MS spectra of **3** and **4** (Supporting Information Figure S1) indicated they were very similar in structure to MC-WA (**2**), except that the fragments attributed to the position four amino acid contained either 14 or 42 Da additional mass (Table 1). The presence of Adda was indicated by mass losses of 134 Da (loss of a portion of the Adda sidechain) and 313 Da (loss of the entire amino acid) [22]. Microcystins commonly contain an 83 Da amino acid in position seven which is frequently Mdha, although this can also be dehydrobutyrine (Dhb). Whilst these isometric moieties cannot be distinguished by mass spectrometry (MS) alone, the reaction rate of a recently developed thiol derivatization technique can be used to identify which amino acid is present [23]. A microcystin containing a terminal alkene, such as that found in Mdha, reacts rapidly with β-mercaptoethanol under alkaline conditions, causing a mass increase of 78 Da [24]. However, the reaction rate is hundreds of times slower for Dhb [23]. The β-mercaptoethanol derivatization of all microcystins detected in the methanol extract of CAWBG11 progressed rapidly and near complete reaction had occurred within two hours (Figure 2). This indicated that all of the microcystins produced by CAWBG11, including MC-WAba and MC-WL, contained Mdha rather than Dhb.

**Table 1:** Positive ion liquid chromatography-tandem mass spectrometry fragment ions for **2**–**4** observed by electrospray ionization collision-induced dissociation.

Fragment Assignment ^a^	MC-WA (2)	MC-WAba (3)	MC-WL (4)
M + H	983	997	1025
M − H_2_O + H	965	979	1007
M − Mdha − H_2_O + H	882	896	
M − Masp or Glu + H	854		896
M − Adda sidechain + H	849	863	891
M − Adda sidechain − H_2_O + H	831	845	873
M − Adda + H	670	684	712
M − Adda − H_2_O + H	652	666	694
Adda-Glu-Mdha-Ala-Trp-Masp − NH_3_ + H	895	895	
Adda-Glu-Mdha-Ala-Trp − NH_3_ + H	766	766	766
Adda-Glu-Mdha-Ala − NH_3_ + H	580	580	580
Adda-Glu-Mdha − NH_3_ + H	509	509	509
Adda′-Glu-Mdha-Ala-Trp + H	632	632	632
Adda-Glu-Mdha-Ala + H	446	446	446
Adda′-Glu-Mdha + H	375	375	375
Mdha-Ala-Trp-Masp-Z + NH_4_	558	572	600
Ala-Trp-Masp-Z + NH_4_	475	489	517
Trp-Masp-Z + NH_4_	404	418	446
Mdha-Ala-Trp-Masp-Z + H	541	555	583
Ala-Trp-Masp-Z + H	458	472	500
Trp-Masp-Z + H	387	401	429

^a^ Z = Position four amino acid, for **2** = 71 Da, **3** = 95 Da and **4** = 113 Da; Adda′ = Adda minus NH_2_ and the sidechain (C_9_H_11_O).

**Figure 3:**
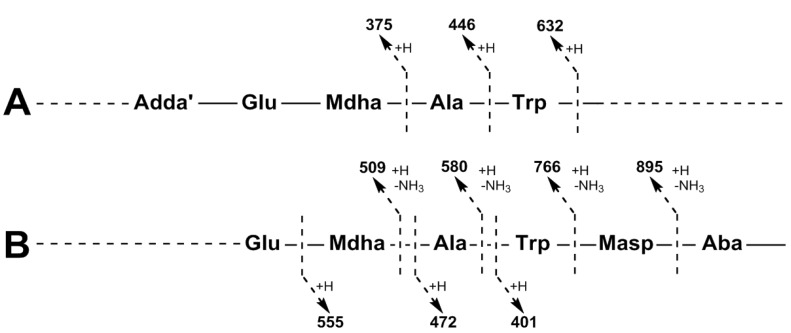
Tandem mass spectrometry fragment ions indicating the amino acid sequence in MC-WAba.

For MC-WAba (**3**), the fragment ion series starting with Adda’-Glu-Mdha (*m/z* 375) was extended to include Ala and Trp (Figure 3A). This sequence was supported by the ion series containing Adda minus NH_3_ (*m/z* 509, 580, 766 and 895), which was extended to include Masp (Figure 3B). A fragment ion series which began with Trp-Masp-Aba (*m/z* 401, Figure 3B) and extended in the opposite direction to include Ala and Mdha gave the complete amino acid sequence of Adda-Glu-Mdha-Ala-Trp-Masp-Aba. A fragment resulting from the loss of Mdha and water (*m/z* 896, Table 1) indicated that Adda and the Aba residue were joined and that the structure was cyclic. The amino acid sequence in MC-WL (**4**) was similarly established. The stereochemistries of **3** and **4** were not determined, but are assumed to be the same as for other microcystins in CAWBG11 characterized by NMR [16].

Several compounds in the extract of *Microcystis* CAWBG11 yielded MS/MS daughter ions resulting from loss of 134 or 313 Da, which represent the loss of Adda and are characteristic of microcystins (Supporting Information Figures S2–S4). These compounds rapidly reacted with β‑mercaptoethanol, as would be expected from a microcystin containing Mdha. However, the compounds had masses (Table 2) which did not correspond to presently described microcystins. Tandem MS analyses indicated that each microcystin contained a high mass amino acid in position two (190, 202 or 218 Da). These high mass amino acids occurred together with arginine (Arg, MC-XRs), alanine (MC-XAs), or aminobutanoic acid (Aba, MC-XAbas) at position four. *Microcystis* CAWBG11 also produces microcystins which contain a position four leucine (MC-XLs), however MC-XL congeners containing the high mass amino acids were not observed in the present study, presumably due to their low abundance.

**Table 2:** Structure, molecular masses and retention times of several of the microcystins found in *Microcystis* CAWBG11.

Microcystin		M*_r_* ^a^ (Da)	R*_t_* ^b^ (min)	X	Z
MC-XR	MC-WR (**1**)	1067.5	7.60	186 Da	Arg
	MC-1071 (**5**)	1071.5	7.45	190 Da	Arg
	MC-1083 (**6**)	1083.5	7.37	202 Da	Arg
	MC-1099 (**7**)	1099.5	7.32	218 Da	Arg
MC-XA	MC-WA (**2**)	982.5	9.54	186 Da	Ala
	MC-986 (**8**)	986.5	9.41	190 Da	Ala
	MC-998 (**9**)	998.5	9.31	202 Da	Ala
	MC-1014 (**10**)	1014.5	9.07	218 Da	Ala
MC-XAba	MC-WAba (**3**)	996.5	10.24	186 Da	Aba
	MC-1000 (**11**)	1000.5	9.80	190 Da	Aba
	MC-1012 (**12**)	1012.5	9.58	202 Da	Aba
	MC-1028 (**13**)	1028.5	9.46	218 Da	Aba
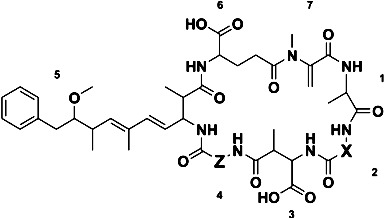					

^a^ Molecular weights are rounded to one decimal place; ^b^ R*_t_* = Retention time on an analytical C_18_ column.

Despite the increased mass of the new microcystins, the congeners were less strongly retained on a reversed-phase C_18_ column than their tryptophan-containing counterparts (Table 2), which indicated that the position two amino acids were slightly more polar than a tryptophan residue. The mass of each of these amino acids corresponded to those of the tryptophan oxidation products; Kyn (190 Da), Oia (202 Da), and Nfk (218 Da; Scheme 1) [8]. The reduced retention times of the new microcystins on reversed-phase C_18_ chromatography (relative to the tryptophan-containing congeners) would also be explained by the presence of an amine in Kyn, a carbonyl in Oia and an amidoketone in Nfk [9]. It was therefore postulated that the new microcystins observed were oxidation products of the tryptophan-containing congeners.

**Figure 4:**
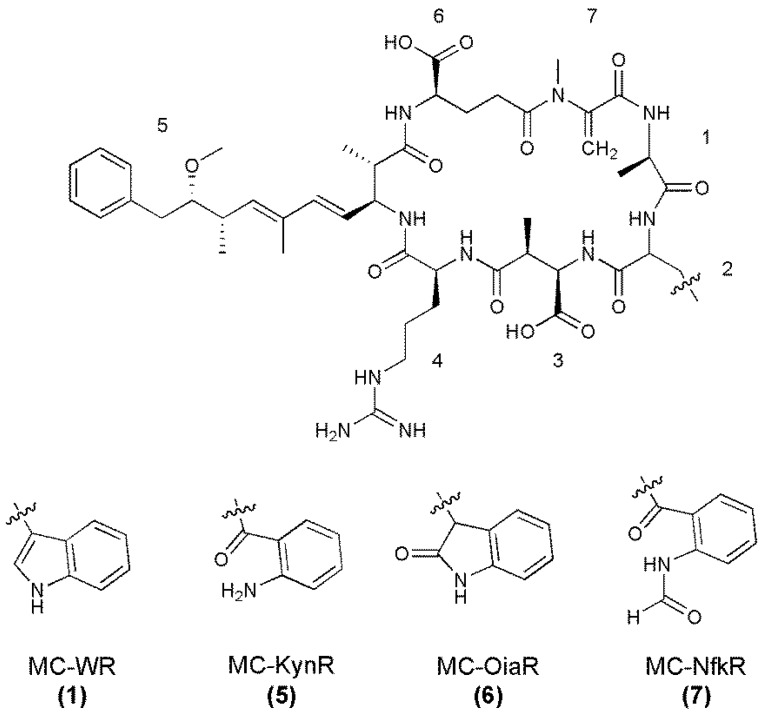
Structures of MC-WR and its oxidized tryptophan congeners, with amino acid numbering (1–7) indicated (Configuration for **5**‑**7** assumed).

The MS/MS spectra of **5**–**7** (Supporting Information Figure S2) indicated that they had structures very similar to MC-WR (**1**), but contained amino acids in position two with additional masses of 4, 16, or 32 Da (Table 3). Due to the masses of the position two amino acids and the reduced retention times observed by reversed-phase C_18_ chromatography, these microcystins were postulated to be MC-KynR (**5**), MC-OiaR (**6**), and MC-NfkR (**7**, Figure 4).

**Table 3:** Liquid chromatography-tandem mass spectrometry fragment ions for **1** and **5**–**7** observed by electrospray ionization collision-induced dissociation.

Fragment Assignment ^a^	MC-WR (1)	MC-KynR (5)	MC-OiaR (6)	MC-NfkR (7)
M + H	1068	1072	1084	1100
M − H_2_O + H	1050	1054	1066	1082
M − Ala + H	997	1001	1013	1029
M − CH_2_NHCN_2_H_3_ + H	996	1000	1012	1028
M − Glu or Masp + H	939	943	955	971
M − Adda sidechain + H	934	938	950	966
M − Adda + H	755	759	771	787
Masp-Arg-Adda − CO + H or Arg-Adda-Glu − CO + H	571	571	571	571
Masp-Arg-Adda-Glu + H	728	728	728	728
Masp-Arg-Adda + H or Arg-Adda-Glu + H	599	599	599	599
Arg-Adda + H	470	470	470	470
Arg-Adda-Glu − NH_3_ + H	582	582	582	582
Arg-Adda − NH_3_ + H	453	453	453	453
Mdha-Ala-X-Masp-Arg + H	626	630	642	658
Ala-X-Masp-Arg + H	543	547	559	575
X-Masp-Arg + H	472	476	488	504
Adda′-Glu-Mdha-Ala + H	446	446	446	446
Adda′-Glu-Mdha + H	375	375	375	375
Mdha-Ala-X-Masp + H	470	474	486	502
Mdha-Ala-X + H	341	345	357	373

^a^ X = Position two amino acid, for **1** = 186 Da, **5** = 190 Da, **6** = 202 Da and **7** = 218 Da; Adda′ = Adda minus NH_2_ and the sidechain (C_9_H_11_O); CH_2_NHCN_2_H_3_ is a fragment of the Arg sidechain.

Fractionation of a *Microcystis* CAWBG11 extract yielded a semi-pure mixture of the oxidized MC-WR congeners that was analyzed by high-resolution electrospray ionization mass spectrometry (HRESIMS) to further investigate the identity of the high mass amino acids. This yielded mass-to-charge ratios which were consistent with the protonated ions of the proposed structures **5**–**7** (Table 4). When the molecular formulae for **5**–**7** were compared to the molecular formula for MC-WR (MH^+^ C_54_H_74_N_11_O_12_), there was a resemblance to the oxidation pattern presented in Scheme 1; where one oxygen was gained to form MC-OiaR, a second oxygen gained to form MC-NfkR and carbon monoxide subsequently lost to form MC-KynR.

**Table 4:** High-resolution electrospray ionization mass spectrometry analysis of a semi-pure mixture of the oxidized MC-WR congeners.

Microcystin	Measured MH^+^	Molecular Formula (MH^+^)	Calculated MH^+^	Deviation (ppm)
MC-WR (**1**)	1068.5465	C_54_H_74_N_11_O_12_	1068.5513	−4.5
MC-OiaR (**6**)	1084.5449	C_54_H_74_N_11_O_13_	1084.5462	−1.2
MC-NfkR (**7**)	1100.5449	C_54_H_74_N_11_O_14_	1100.5411	+3.4
MC-KynR (**5**)	1072.5431	C_53_H_74_N_11_O_13_	1072.5462	−2.9

**Figure 5:**
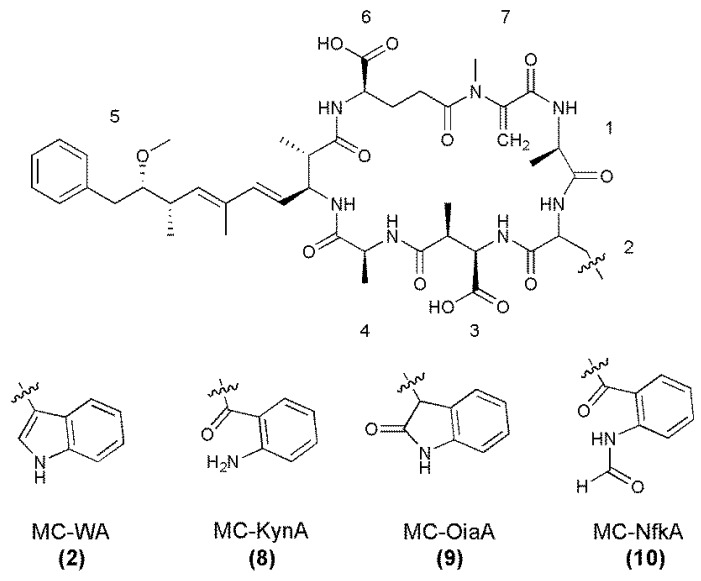
Structures of MC-WA and its oxidized tryptophan congeners, with amino acid numbering (1–7) indicated (Configuration for **8**‑**10** assumed).

The MS/MS spectra of **8**–**10** (Supporting Information Figure S3) indicated that they were microcystins very similar to MC-WA in structure, but contained position two amino acids with additional masses of 4, 16, or 32 Da, respectively (Table 5). As with the arginine-containing microcystins, these were postulated to be Kyn (MC-KynA, **8**), Oia (MC-OiaA, **9**), and Nfk (MC-NfkA, **10**, Figure 5). The HRMS analysis of **8**–**10** yielded *m/z* 1009.4670, 1021.4634, and 1037.4598, consistent with sodium adduct ions of the proposed structures **8**–**10** (Supporting Information Table S1).

**Table 5:** Liquid chromatography-tandem mass spectrometry fragment ions for **2** and **8**–**10** observed by electrospray ionization collision-induced dissociation.

Fragment Assignment ^a^	MC-WA (2)	MC-KynA (8)	MC-OiaA (9)	MC-NfkA (10)
M + H	983	987	999	1015
M − H_2_O + H	965	969	981	997
M − Mdha − H_2_O + H	882	886	898	914
M − Adda sidechain + H	849	853	865	881
M − Adda sidechain − H_2_O + H	831	835	847	863
M − Adda + H	670	674		702
M − Adda − H_2_O + H	652	656	668	684
Adda-Glu-Mdha-Ala-X-Masp − NH_3_ + H	895	899	911	927
Adda-Glu-Mdha-Ala-X − NH_3_ + H	766	770	782	798
Adda-Glu-Mdha-Ala − NH_3_ + H	580	580	580	580
Adda-Glu-Mdha − NH_3_ + H	509	509	509	509
Adda′-Glu-Mdha-Ala-X + H	632	636	648	664
Adda′-Glu-Mdha-Ala + H	446	446	446	446
Adda′-Glu-Mdha + H	375	375	375	375
Mdha-Ala-X-Masp-Ala + H	541	545	557	573
Ala-X-Masp-Ala + H	458	462	474	490
X-Masp-Ala + H	387	391		419
Mdha-Ala-X + H	341	345	357	373

^a^ X = Position two amino acid, for **2** = 186 Da, **8** = 190 Da, **9** = 202 Da and **10** = 218 Da; Adda′ = Adda minus NH_2_ and the sidechain (C_9_H_11_O).

**Figure 6:**
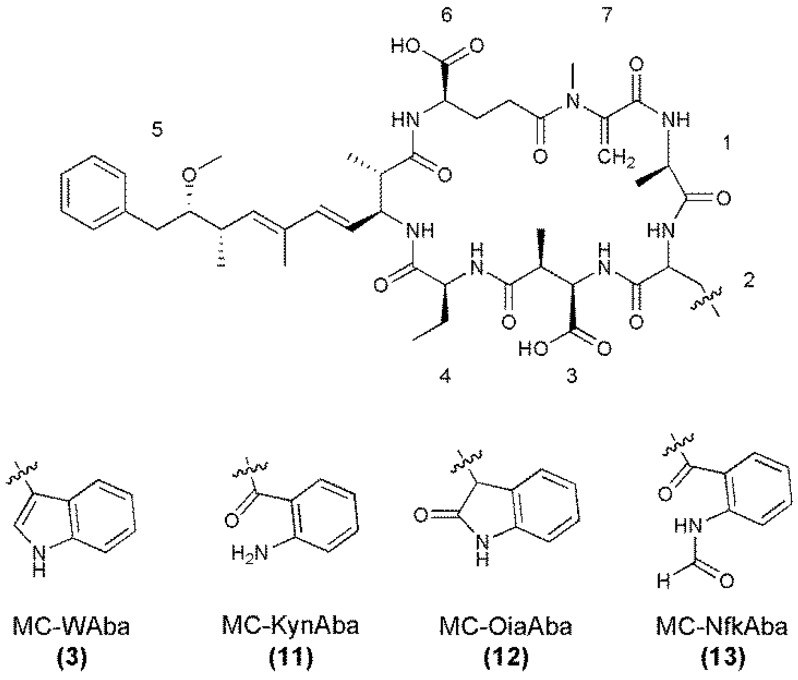
Structures of MC-WR and its oxidized tryptophan congeners, with amino acid numbering (1–7) indicated (Configuration assumed).

Since the MC-XAba microcystins were present in low quantities, the only indication of the presence of MC-KynAba (**11**) (Figure 6) was an MH^+^ ion at *m*/*z* 1001.5. The MS/MS spectra of **12** and **13** (Supporting Information Figure S4) indicated that they were microcystins very similar in structure to MC-WAba, but containing an amino acid at position two with additional mass of 16 or 32 Da (Table 6). Due to their mass and reduced reversed-phase C_18_ retention times, these amino acids were postulated to be Oia (MC-OiaAba; **12**) and Nfk (MC-NfkAba; **13**; Figure 6). The low abundance of the MC-XAba congeners also prevented further purification and HRMS of **12** and **13**.

**Table 6:** Liquid chromatography-tandem mass spectrometry fragment ions for **3**, **12**, and **13** observed by electrospray ionization collision-induced dissociation.

Fragment Assignment ^a^	MC-WAba (3)	MC-OiaAba (12)	MC-NfkAba (13)
M + H	997	1013	1029
M − H_2_O + H	979	995	1011
M − Mdha − H_2_O + H	896		928
M − Aba − H_2_O + H		910	
M − Glu or Masp − H_2_O + H	850	866	878
M − Adda sidechain + H	863	879	895
M − Adda sidechain − H_2_O + H	845	861	877
M − Adda + H	684	700	716
M − Adda − H_2_O + H	666		698
Adda-Glu-Mdha-Ala-X-Masp − NH_3_ + H	895	911	
Adda-Glu-Mdha-Ala-X − NH_3_ + H	766	782	798
Adda-Glu-Mdha-Ala − NH_3_ + H	580	580	580
Adda-Glu-Mdha − NH_3_ + H	509	509	509
Adda′-Glu-Mdha-Ala-X + H	632	648	664
Adda′-Glu-Mdha-Ala + H	446	446	446
Adda′-Glu-Mdha + H	375	375	375
Mdha-Ala-X-Masp-Aba + H	555	571	587
Ala-X-Masp-Aba + H	472		504
X-Masp-Aba + H	401	417	433

^a^ X = Position two amino acid, for **3** = 186 Da, **12** = 202 Da and **13** = 218 Da; Adda′ = Adda minus NH_2_ and the sidechain (C_9_H_11_O).

### 2.2. Nuclear Magnetic Resonance Spectroscopy of MC-NfkA

**Figure 7:**
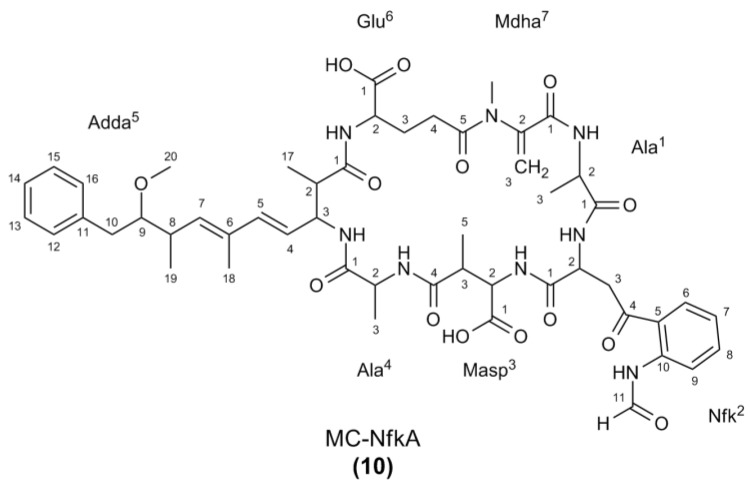
Structure of MC-NfkA (**10**) with atom numbering used for the nuclear magnetic resonance spectroscopy assignment.

MC-NfkR (**7**) and MC-NfkA (**10**) (Figure 7) were the most abundant of the oxidized tryptophan microcystins. Whilst **7** was not able to be separated from MC-LR (produced in high levels by *Microcystis* CAWBG11) using the current fractionation procedure, a sufficient quantity of **10** was purified to verify its structure using NMR spectroscopy.

Interpretation of COSY, ROESY, HSQC, and HMBC spectra (Supporting Information Figures S5–S11) allowed assignment of the ^1^H and ^13^C signals (Table 7). Many of the signals observed were similar to those reported for MC-WA (**2**) [16], except that the signals indicative of the tryptophan residue were absent. Instead, there were signals in the ^1^H NMR spectrum consistent with an Nfk residue [25]; an asymmetrically disubstituted aromatic ring with proton resonances *ca.* 0.5 ppm downfield from those observed in tryptophan [δ 8.06 (d; H6), 7.24 (dd; H7), 7.49 (dd; H8), and 8.49 (d; H9)] and a singlet at 8.43 ppm (H11) arising from a formamide moiety. As the H11 signal in **10** resonated as a singlet, the *N*-formyl moiety was determined to be in the *cis*-configuration [26].

The remainder of the NMR assignment was consistent with that of a microcystin following the general structure and containing an alanine in the second variable position (four). The presence of the unusual amino acid Adda was indicated by signals in the ^1^H NMR spectrum for a monosubstituted aromatic ring [δ 7.19 (2H, d, H12/16), 7.24 (2H, dd, H13/15), and 7.16 (t, H14)], a trisubstituted diene system [δ 5.58 (dd, H4), 6.24 (d, H5), and 5.41 (d, H7)], a methoxyl group [δ 3.24 (3H, s, H20)] and a downfield methyl signal [δ 1.64 (3H, s, H18)] [27]. The diene system was shown to be in the 4(*E*),6(*E*)-configuration, due to the large coupling constant between the H4 and H5 signals (15.5 Hz) and the H5–H7 correlation observed in the ROESY NMR spectrum (Table 6) [28].

The geminal alkene proton signals observed at 5.71 ppm (s, H3A) and 5.27 ppm (s, H3B), and a *N*‑methyl signal at 3.28 ppm were consistent with the presence of Mdha [15,29]. Signals representative of two consecutive methylene groups [δ 1.93 (m, H3A), 2.22 (m, H3B), 2.48 (m, H4A), and 2.66 (m, H4B)] attached to a downfield methine [δ 4.06 (m, H2)] were consistent with the *iso*-glutamic acid residue commonly observed in position six of microcystins [30]. The presence of *iso*-Masp which is frequently found in position three of microcystins, was confirmed due to a methine proton coupled to a methyl group and a further (downfield) methine proton [δ 4.36 (m; H2), 3.14 (m, H3), and 1.06 (3H, d, H5)] [31]. 

Finally, the ^1^H NMR spectrum contained two sets of downfield methine signals coupled to methyl protons [δ 4.41 (m, H2), 1.26 (3H, d, H3) and δ 4.54 (m, H2), 1.40 (3H, d, H3)], which confirmed the presence of two alanine residues in **10**. Several correlations in the ROESY spectrum between the Adda amide and proton signals from the alanine residue with the more upfield methyl proton resonance (δ 1.26) placed that alanine in position four. The alanine with the more downfield methyl proton resonance (δ 1.40) was therefore attributed to the position one alanine.

Correlations observed in the ROESY NMR spectrum (Supporting Information Figure S11) confirmed the amino acid connectivity indicated by the LC-MS/MS fragmentation data (Table 5) and were consistent with the stereochemistry shown in Figure 5.

**Table 7:** Nuclear magnetic resonance spectroscopy assignments for MC-NfkA in CD_3_OH.

Position ^a^		δ_C_	δ_H_ (*J* in Hz)	COSY	HMBC ^b^	ROESY
**Ala^1^**	1	175.7				
	2	49.9	4.54, m	Ala^1^-3, NH	Ala^1^-1	Ala^1^-3
	3	17.1	1.40, d (7.3)	Ala^1^-2	Ala^1^-1, 2	Ala^1^-2
	NH	-	8.22, d (8.5)	Ala^1^-2		
**Nfk^2^**	1	Nd				
	2	55.9	4.26, m	Nfk-3, NH		
	3	29.1	2.02 (2H), m	Nfk-2		
	4	Nd				
	5	124.9				
	6	132.4	8.06, d (7.2) ^d^	Nfk-7	Nfk-8, 10	
	7	125.5	7.24, dd (7.2, 8.6) ^d^	Nfk-6, 8		Masp-NH
	8	134.6	7.49, dd (8.0, 8.6)	Nfk-7, 9		
	9	122.2	8.49, d (8.0)	Nfk-8	Nfk-5	
	10	139.5				
	11	162.5	8.43, s		Nfk-10	Nfk-CHO-NH
	CHO-NH	-	8.93, br s			Nfk-11
	NH	-	8.32, d (6.8)	Nfk-2		
**Masp^3^**	1	Nd				
	2	58.5	4.36, m	Masp-3, NH		Masp-5
	3	42.3	3.14, m	Masp-2, 5		Masp-5; Ala^4^-NH
	4	177.8	-			
	5	15.8	1.06, d (6.9)	Masp-3	Masp-2, 3, 4	Masp-2, 3
	NH	-	8.06, d (8.3)	Masp-2		
**Ala^4^**	1	172.5	-			
	2	49.0 *^c^*	4.41, m	Ala^4^-3, NH	Ala^4^-1	Ala^4^-3; Adda-NH
	3	17.5	1.26, d (7.3)	Ala^4^-2	Ala^4^-1, 2	Ala^4^-2, NH; Adda-NH
	NH	-	8.72, d (9.2)	Ala^4^-2		Ala^4^-3; Adda-NH; Masp-3
**Adda^5^**	1	176.5	-			
	2	44.6	3.16, m	Adda-3, 17		Glu-NH
	3	56.7	4.57, m	Adda-2, 4, NH	Adda-17	Adda-17, NH
	4	127.3	5.58, dd (9.3, 15.5)	Adda-3, 5	Adda-5, 6	Adda-18, NH
	5	138.6	6.24, d (15.5)	Adda-4	Adda-3, 6, 7, 18	Adda-7
	6	136.0	-			
	7	136.4	5.41, d (10.0)	Adda-8, 18	Adda-5, 6, 8, 9	Adda-5, 19
	8	37.7	2.59, m	Adda-7, 9, 19	Adda-6	Adda-19, 20
	9	88.4	3.25, m ^c^	Adda-8, 10A, 10B	Adda-11, 19, 20	Adda-19
	10	39.3	2.82, dd (4.7, 14.0)	Adda-9, 10B	Adda-8, 9, 11, 12/16	Adda-10B, 12/16, 19
			2.68, dd (7.4, 14.0)	Adda-9, 10A	Adda-8, 9, 11, 12/16	Adda-10A, 12/16, 19
	11	140.3	-			
	12/16	130.1	7.19, d (7.8)	Adda-13/15	Adda-10, 14	Adda-10A, 10B
	13/15	128.7	7.24, dd (7.4, 7.8)	Adda-12/16, 14	Adda-11	
	14	126.9	7.16, t (7.4)	Adda-13/15	Adda-12/16	
	17	15.9	1.07, d (6.7)	Adda-2	Adda-1, 3	Adda-3
	18	12.9	1.64, s	Adda-7	Adda-6, 7	Adda-4
	19	16.4	1.00, d (6.9)	Adda-8	Adda-7, 8, 9	Adda-7, 8, 9, 10A, 10B
	20	58.7	3.24, s		Adda-9	Adda-8
	NH	-	8.01, d (9.2)	Adda-3		Adda-3, 4; Ala^4^-2, 3, NH
**Glu^6^**	1	Nd				
	2	56.5	4.06, m	Glu-3A, 3B, NH		
	3	29.7	2.22, m	Glu-2, 4A, 4B		Glu-3-B
			1.93, m	Glu-2, 4A, 4B		Glu-3A
	4	33.5	2.66, m	Glu-3A, 3B, 4B		Glu-4B
			2.48, m	Glu-3A, 3B, 4A		Glu-4A
	5	177.1				
	NH	-	8.39, d (7.2)	Glu-2		Adda-2
**Mdha^7^**	1	165.9				
	2	146.3				
	3	113.6	5.71, s	Mdha-3B	Mdha-1	Mdha-3B
			5.27, s	Mdha-3A	Mdha-1, 2	Mdha-3A
	*N*-CH_3_	38.2	3.28, s ^c^		Mdha-2; Glu-5	

^a^ Position in structure indicated by the superscript number; nd = Not detected; br = Broad signal; s = Singlet; d = Doublet; t = Triplet; m = Multiplet; dd = Doublet of doublets. ^b^ HMBC were optimized for 10 Hz and are from the proton(s) stated to the indicated carbon. ^c^ Signals were overlapped and calibrated using the 2-D HSQC spectrum. ^d^ Multiplicity and coupling constants were determined using 1-D selective TOCSY experiments.

### 2.3. Oxidation of Tryptophan-Containing Microcystins

In order to determine whether oxidized tryptophan microcystins could be produced via oxidation of existing tryptophan microcystins, an extract of *Microcystis* CAWBG11 was shielded from light and exposed to atmospheric oxygen, during which time, sub-samples were analyzed periodically by LC-MS (Supporting Information Figure S12a). After 124 h, the level of tryptophan-containing microcystins (for example, MC-WA) had decreased by only 4%. In turn, the level of the oxidized tryptophan microcystins (for example, MC-NfkA) had increased. Stirring at *ca.* 250 rpm (to increase dissolved oxygen concentrations) did not have any effect on the rate of tryptophan oxidation (Supporting Information Figure S12b). Addition of an oxidizing agent (hydrogen peroxide) did increase the rate of tryptophan oxidation and after 124 h, the level of oxidized tryptophan microcystins had increased by *ca.* 28% (Supporting Information Figure S12c). As tryptophan-containing microcystins were shown to be converted into oxidized tryptophan congeners through exposure to oxidizing agents, it is probable that during the purification of **10**, some MC-WA was oxidized to form MC-NfkA. As with other microcystin congeners, it is very likely that these oxidation artifacts will inhibit protein phosphatases 1 and 2A and pose a health threat to humans and animals. Unfortunately, the material isolated for structural characterization was insufficiently pure to proceed with toxicology or protein phosphatase inhibition studies at the present time. LC-MS^2^ analysis showed that pure standards of MC-WR and MC-LW were similarly oxidized to MC-WNfk and MC-LNfk by exposure to H_2_O_2_, and that trace amounts of these oxidized congeners were detectable in the standards that had been stored in methanol for several months at −20 °C.

### 2.4. Presence of Intracellular Oxidized Tryptophan Microcystins

**Figure 8:**
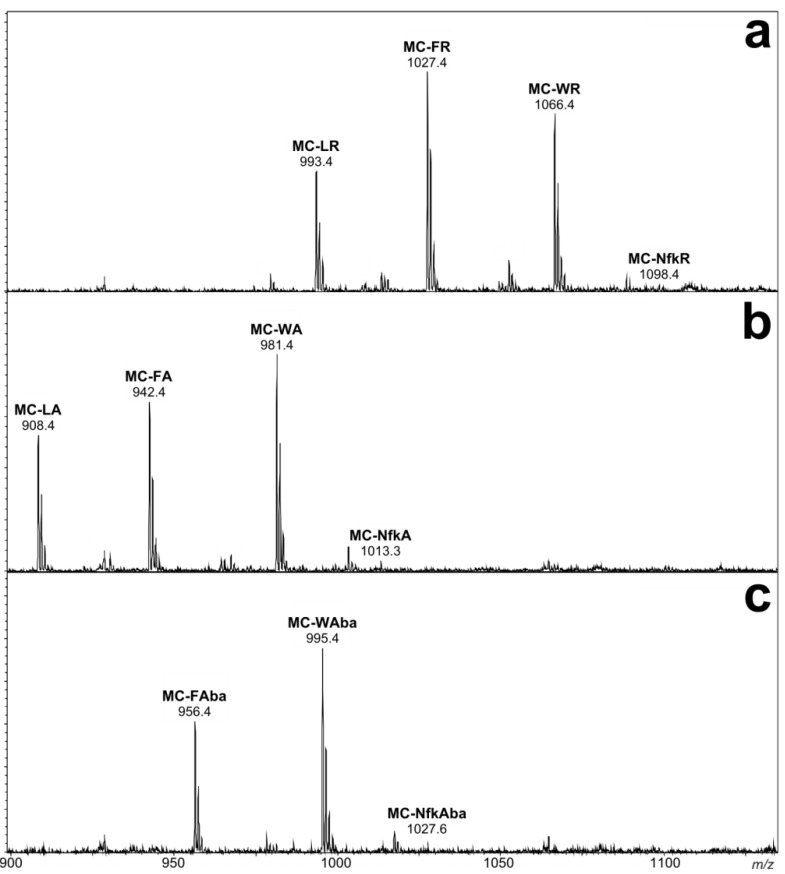
Negative ion liquid chromatography-mass spectra of an extract of CAWBG11 performed under mild (extraction) conditions. Spectra focusing on (**a**) the -XR congeners, (**b**) the -XA congeners and (**c**) the -XAba congeners.

In order to determine whether oxidized tryptophan microcystin congeners were present inside cyanobacteria, a healthy culture of *Microcystis* CAWBG11 was harvested under mild conditions and in a short time period. LC-MS analysis of the extract revealed the presence of conventional microcystins produced by CAWBG11 as well as low levels of Nfk-containing microcystins (Figure 8).

During the previous experiment, the oxidation of tryptophan-containing microcystins into Kyn-, Oia- and Nfk-containing microcystins was evident after a long time period (124 h). Over a short period of time (2 h), tryptophan-oxidation was only detected when hydrogen peroxide was present (Supporting Information Figure S12). As the mild extraction above was completed in less than two hours, the oxidized tryptophan microcystins observed were most likely present inside the cells. It is not clear how intracellular oxidized tryptophan microcystins are produced in cyanobacteria. Whilst there is a possibility that Kyn, Oia, and Nfk could be incorporated into the structure by a microcystin synthase, it is more likely that the oxidation occurs post-synthesis. Whether the oxidation is due to natural oxygen levels, reactive oxygen species or is enzymatically mediated, remains to be elucidated.

### 2.5. Implications of These Findings

Whilst oxidized tryptophan residues have been noted in polypeptides [8,9,10,11], their presence in microcystins has not. LC-MS/MS analysis of eight unidentified compounds in CAWBG11 indicated the presence of microcystins containing position two amino acids with the mass of known tryptophan oxidation products (Kyn, Oia and Nfk). A sufficient quantity of one of the microcystins (MC-NfkA) was purified for characterization by NMR spectroscopy, which verified the presence of Nfk at position two of the microcystin.

It is likely that some of these oxidized tryptophan microcystin congeners have been encountered in the past, as mass-to-charge ratios consistent with MC-OiaR and/or MC-NfkR have been reported previously [32,33]. In both of these studies, MC-WR was present in the samples, making it likely that oxidized tryptophan microcystins would also have been present. The findings of the present study will now enable researchers working with samples of tryptophan-containing microcystins to assign previously unidentified oxidized analogs.

The oxidation of tryptophan-containing microcystins poses an additional concern for researchers quantifying microcystins in samples that include tryptophan-containing congeners. Whilst the rate of oxidation under normal storage conditions is unknown, we observed that during prolonged storage of tryptophan-containing microcystin standards (MC-WR and MC-LW), some of the microcystin became oxidized. This would result in a lower than expected signal from samples and standards, causing inaccurate measurements. A previous study investigating the oxidation of tryptophan residues in proteins found that the addition of free-radical scavengers (free tryptophan, pyridoxine or Trolox) protected proteinaceous tryptophan residues [34]. However, since tryptophan oxidation has also been shown to be catalyzed by exposure to light [35] and transition metals [36], further investigation of these parameters would also be of benefit.

## 3. Experimental Section

### 3.1. General Experimental Procedures

NMR spectra were recorded on a Bruker AVIII-600 NMR spectrometer equipped with a TCI cryoprobe and Z-gradient coils operating at 600 MHz for ^1^H and 150 MHz for ^13^C. Chemical shifts were determined at 298 K and are reported relative to the solvent signal (C*H*D_2_OH; ^1^H 3.31 ppm, *C*D_3_OH ^13^C 49.0 ppm). HRESIMS was performed on a Bruker MicrOTOF mass spectrometer. LC-MS and LC-MS/MS analyses were performed on a Bruker AmaZon X ESI mass spectrometer coupled to a Dionex UltiMate 3000 HPLC system. Reversed-phased C_18_ separations were conducted using YMC-gel ODS-A (YMC) and size exclusion chromatography was conducted using Sephadex LH-20 (Pharmacia Fine Chemicals). HPLC purification was performed using Waters 515 HPLC pumps coupled to a photodiode array detector (200–400 nm; Waters 2996) and an Econosil C_18_ Column (250 × 10 mm, 10 µm; Alltech).

### 3.2. Liquid Chromatography-Mass Spectrometry Analysis

Samples (20 µL) for LC-MS and LC-MS/MS were separated on a C_18_ column (Ascentis Express C_18_, 100 × 2.1 mm, 2.7μm; Supleco Analytical) at a flow of 200 µL/min using a gradient of 2% solvent A (acetonitrile + 0.1% formic acid, v/v) and 98% solvent B (acetonitrile + 0.1% formic acid, v/v) with the following gradient program; the sample was loaded in 10% B; 10% B was held for 1 min and increased to 100% B over 12 min; 100% B was held for 2 min; the solvent composition was returned to 10% B in 1 min and the column re‑equilibrated for 4 min. The eluting compounds were ionized using a capillary voltage of 3.5 kV and a nebulizer pressure of 3.0 bar. Desolvation was accomplished with a nitrogen flow of 8 L/min at 220 °C. Mass spectra were acquired for positive or negative ions over a range of *m/z* 100–2000. Daughter ion scans were obtained from the singly-protonated ions of the target compounds by collision-induced dissociation (collision amplitude of 1.0).

### 3.3. β-Mercaptoethanol Derivatization for Mdha/Dhb Determination 

A recently developed thiol derivatization technique [23] was used to determine which of the isometric amino acids; Mdha or Dhb, was present in CAWBG11 microcystins. A methanol extract of CAWBG11 (1.42 mL) was mixed with 200 mM NaHCO_3_ (pH 9.7; 360 µL) in a septum-capped vial and left to equilibrate at 30 °C. Following LC-MS analysis of the original extract, β‑mercaptoethanol (20 µL) was added to the extract and the vial inverted to mix. The reaction mixture was maintained at 30 °C in the sample tray of the LC-MS and injections were made periodically over a 6 h period.

### 3.4. Isolation of the MC-XA Oxidized Tryptophan Congeners

*Microcystis* sp. CAWBG11 was isolated from a bloom sample obtained from Lake Hakanoa (Huntly, New Zealand) in 2005 [37]. The culture is maintained alive and cryopreserved in the Cawthron Institute micro-algae culture collection [38]. The 16S ribosomal RNA gene partial sequence and full 16S-23S rRNA intergenic spacer sequence are available on GenBank (EF634465).

*Microcystis* sp. CAWBG11 was grown in 20 × 20 L plastic carboys, each containing 16 L of MLA media [39]. Cultures were grown at 18 °C under a 12:12 h light/dark cycle with a photon-flux of 100 µE m^−2^ s^−1^. After 40 days, the cultures were harvested using plankton netting (11 µm mesh). The concentrated cell material was lyophilized and stored at −20 °C until extracted.

Freeze-dried cells (76.9 g) were extracted in 7:3 EtOH/H_2_O (5 × 800 mL). The remaining cell pellet was extracted in MeOH (5 × 250 mL). A voucher of the cellular material extracted (JP2-033-05) is held at the Department of Chemistry, University of Waikato, Hamilton, New Zealand. The crude extracts (5.6 g and 0.45 g, respectively) were evaporated and individually fractionated by reversed-phase C_18_ chromatography (50 g) using a steep stepped gradient from water to MeOH to DCM, where **5**–**10** eluted between 3:7 and 1:1 MeOH/H_2_O. These fractions were combined (331.8 mg) and separated on a reversed-phase C_18_ column (20 g) acidified with 0.1% formic acid (FA; v/v) using a steep stepped gradient from acidified water to acidified MeOH to MeOH to DCM, where **5**–**7** eluted with 3:2 MeOH/H_2_O + 0.1% FA (v/v) and **8**−**10** eluted with 7:3 MeOH/H_2_O + 0.1% FA (v/v).

The fraction containing **5**–**7** (81.7 mg) was neutralized with K_2_CO_3_ (40 mg) in MeOH (0.6 mL) and separated on a reversed-phase C_18_ column (20 g) using a stepped gradient from water to MeOH. The fraction from this column which eluted in MeOH/H_2_O (3:7; 9.8 mg) was dissolved in MeOH and subjected to size exclusion chromatography to yield a mixture of MC-LR and **5**–**7** (5.6 mg), which was separated by isocratic HPLC using ACN:10 mM ammonium acetate (1:3). The dried samples were lyophilized then residual ammonium acetate was removed by passing the sample (dissolved in 10% MeOH; v/v), through a plug of C_18_ material (200 mg) and eluting with 70% MeOH (v/v) to yield **5** (<0.1 mg), **6** (<0.1 mg), and a mixture of MC-LR and **7** (2.4 mg).

The fraction containing **8**–**10** (127.5 mg) was neutralized with K_2_CO_3_ (40 mg) in MeOH (0.6 mL) and separated on a reversed-phase C_18_ column (20 g) using a stepped gradient from water to MeOH. The fraction from this column which eluted in MeOH/H_2_O (1:3; 34.2 mg) was dissolved in MeOH and subjected to size exclusion chromatography to yield three mixtures with varying proportions of MC-LA, MC-FA, **2** and **8**–**10**. These mixtures (4.9 mg, 6.7 mg and 10.2 mg) were individually fractionated by isocratic HPLC using ACN:10 mM ammonium acetate (27:73) to yield a mixture of MC-LA and **8**–**10** (1.9 mg), which was dissolved in MeOH and subject to repeated size exclusion chromatography to yield a mixture of **8**,**9** (0.1 mg) and a mixture of MC-LA and **9**,**10** (0.6 mg). The mixture containing **10** was finally fractionated by isocratic HPLC using ACN/10 mM ammonium acetate (27:23). The dried sample was lyophilized and residual ammonium acetate removed by passing the sample, dissolved in 10% MeOH (v/v), through a plug of C_18_ material (200 mg) and eluting with 70% MeOH (v/v) to yield **9** (<0.1 mg) and **10** (0.4 mg).

**MC-NfkA** (**10**): White amorphous solid (0.4 mg, 5.20 × 10^−4^%); ^1^H and ^13^C NMR data (CD_3_OH) see Table 6; HRESIMS *m/z* 1037.4598 (calculated for C_51_H_66_N_8_O_14_Na, 1037.4591, Δ +0.8 ppm).

### 3.5. Mild Extraction of Microcystis CAWBG11

A healthy culture of CAWBG11 (150 mL) grown at 20 °C 12:12 h light/dark with no perturbation was harvested on nylon net (100 µm mesh). The concentrated cells were washed with MLA medium (3 × 50 mL; at ambient temperature) then sonicated (35 W, 30 min) in 70% MeOH (25 mL; v/v; previously degassed by sonication). The resulting extract was filtered through nylon net (100 µm mesh) to remove large cellular debris. An aliquot (0.5 mL) was transferred to a microcentrifuge tube, diluted with H_2_O (0.5 mL) and centrifuged (14,000 rcf, 5 min). The supernatant (0.9 mL) was transferred to a septum-capped LC vial and analyzed by LC-MS. This process was completed in 50 min.

### 3.6. Oxidation of Tryptophan-Containing Microcystins

Three aliquots of the CAWBG11 extract described above (7 mL each) were transferred to Falcon tubes and centrifuged (2850 rcf, 10 min). Two aliquots of the supernatant (5 mL) were diluted with Milli-Q water (5 mL each; degassed by sonication) and a third was diluted with 10% hydrogen peroxide (5 mL; degassed by sonication). An aliquot (1 mL) was centrifuged (14,000 rcf, 5 min), before the supernatant (0.9 mL) was analyzed by LC-MS. As this process took *ca.* 10 min to complete, no zero time-point was recorded. Each of the extracts were shielded from light using tin foil, and left at ambient temperature. The first extract diluted with water was stirred (*ca.* 250 rpm), whilst the second extract diluted with water had no further treatment. The extract diluted with hydrogen peroxide was not stirred. Samples (1 mL) were analyzed by LC-MS at 1, 2, 6, 26, 52, and 124 h.

## 4. Conclusions

Tandem MS analysis of unknown microcystins in *Microcystis* CAWBG11 was used to tentatively identify two new tryptophan-containing congeners (MC-WAba and MC-WL). Further LC-MS/MS and HRMS analyses led to the identification of microcystins containing the known oxidation products of tryptophan; Kyn, Oia and Nfk. One of the oxidized tryptophan microcystins (MC-NfkA) was purified in sufficient quantity to confirm its structure by NMR spectroscopy. This resulted in the characterization of ten new microcystin analogs and is the first report of microcystins containing oxidation products of tryptophan. Caution should be taken by researchers working with tryptophan-containing microcystin samples to ensure that they monitor the levels of the tryptophan oxidation products.

## Supplementary Files

Supplementary File 1 Supplementary Materials (PDF, 746 KB)
