# Supplementary material for: Structural Characterization of New Microcystins Containing Tryptophan and Oxidized Tryptophan Residues
Source: Mar Drugs. 2013 Aug 21;11(8):3025–45. doi: 10.3390/md11083025 (PMC3766880; doi:10.3390/md11083025)

Supplementary Materials

Figure S1. Electrospray ionization collision-induced dissociation MS/MS spectra for (**a**) MC-WR (**1**), (**b**) MC-WA (**2**), (**c**) MC-WAba (**3**) and (**d**) MC-WL (**4**).

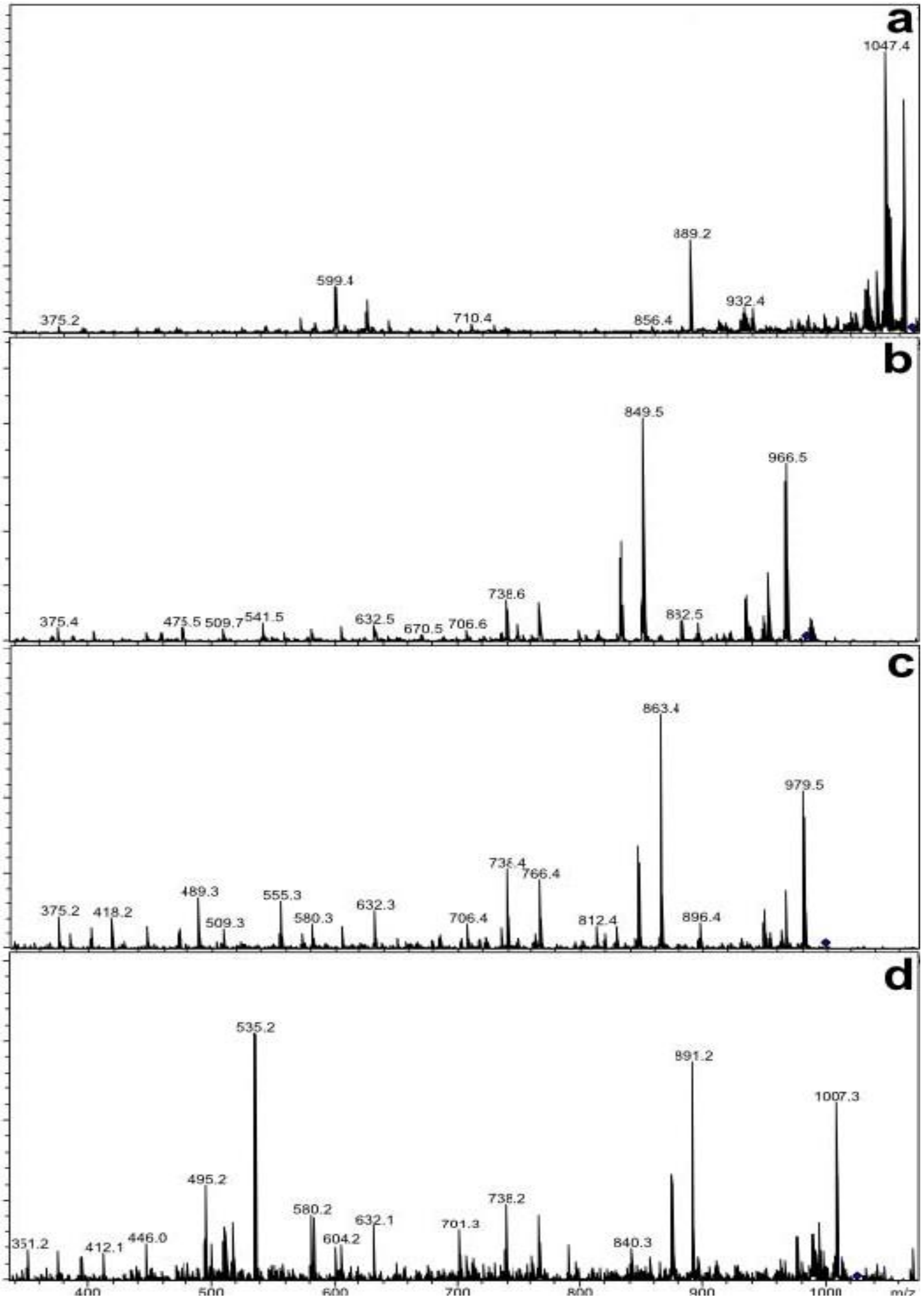

Figure S2. Electrospray ionization collision-induced dissociation MS/MS spectra for (**a**) MC-KynR (**5**), (**b**) MC-OiaR (**6**) and (**c**) MC-NfkR (**7**).

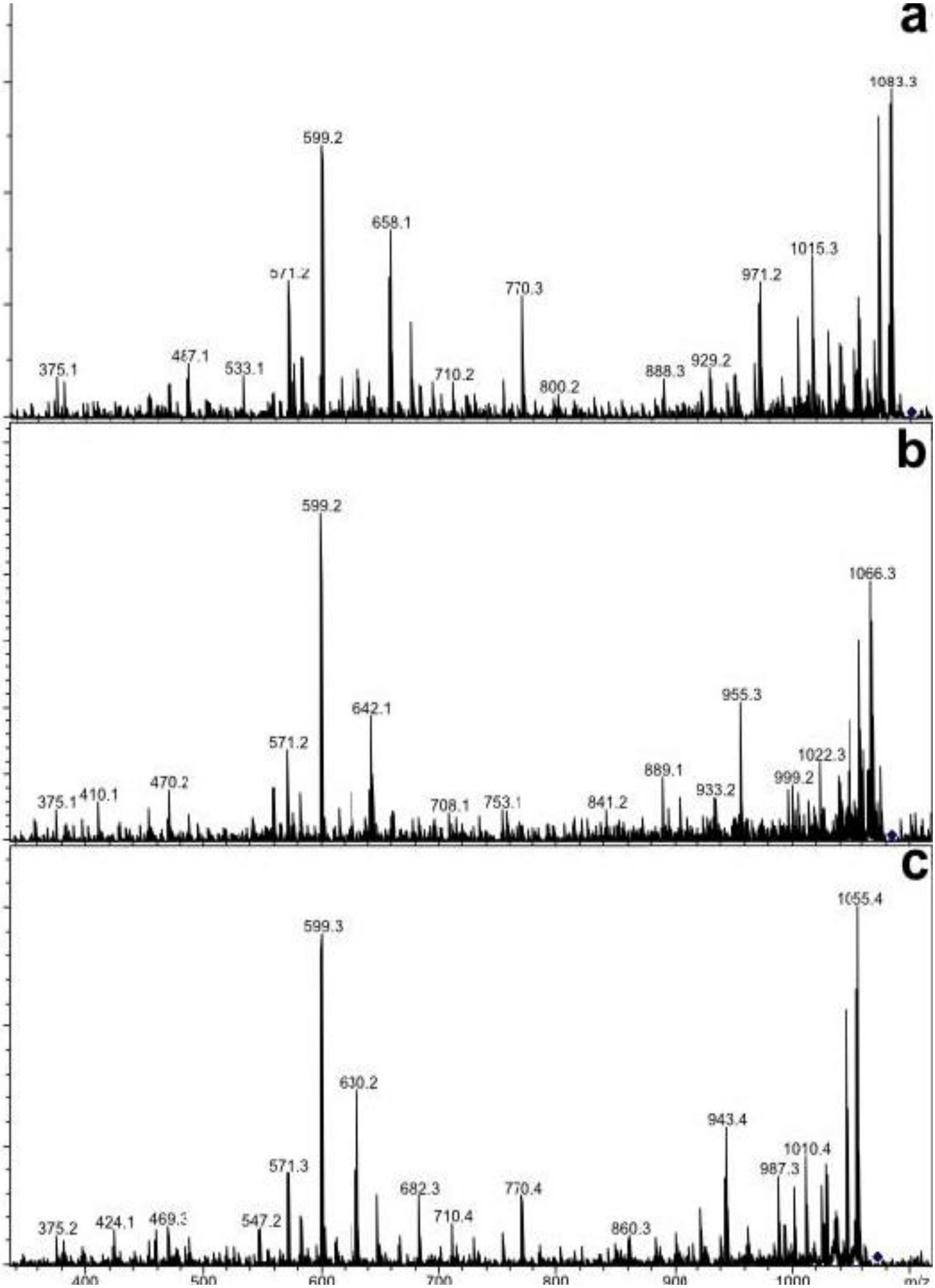

Figure S3. Electrospray ionization collision-induced dissociation MS/MS spectra for (**a**) MC-KynA (**8**), (**b**) MC-OiaA (**9**) and (**c**) MC-NfkA (**10**).

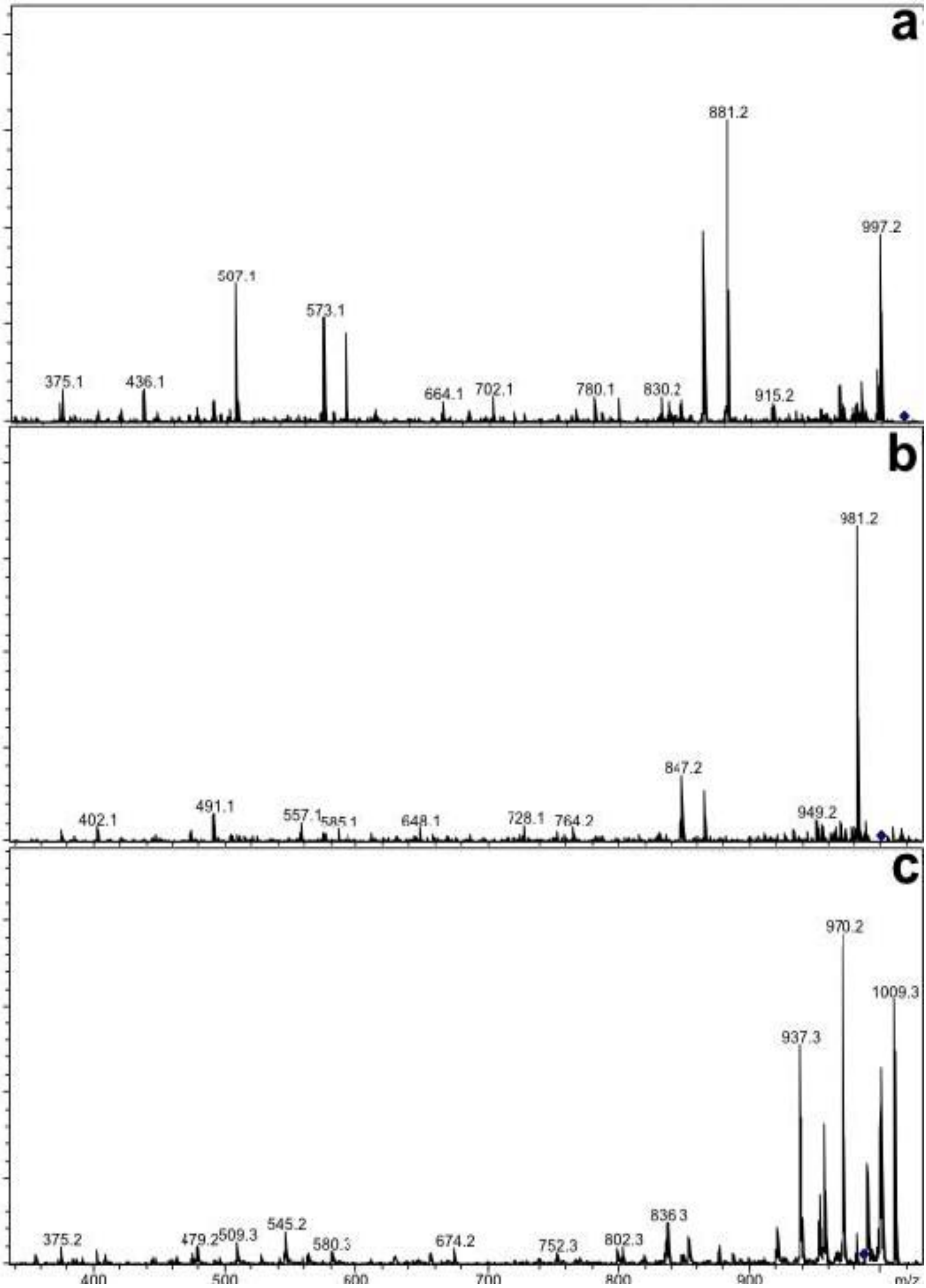

Figure S4. Electrospray ionization collision-induced dissociation MS/MS spectra for (**a**) MC-OiaAba (**12**) and (**b**) MC-NfkAba (**13**).

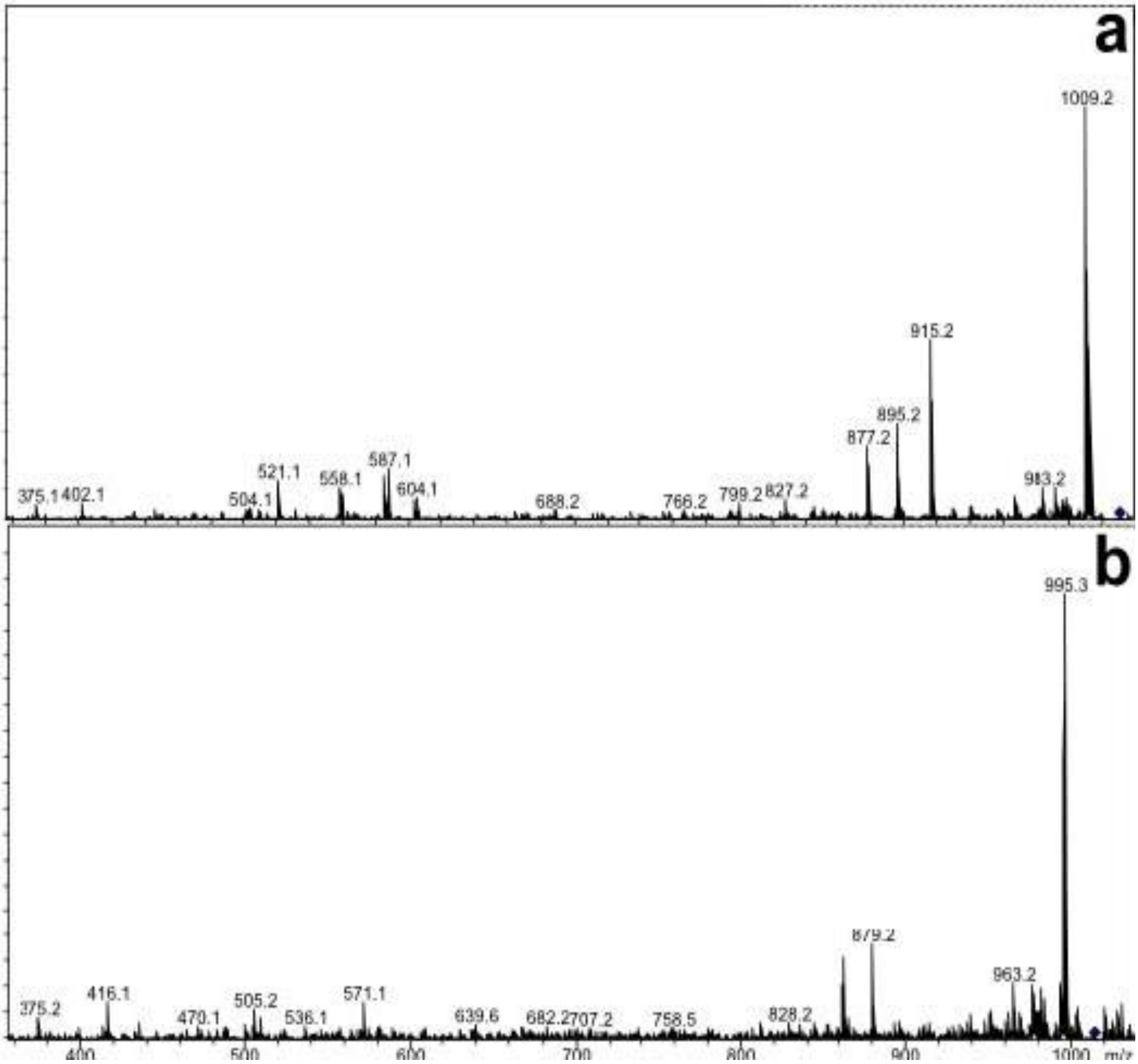

Table S1. High-resolution electrospray ionization mass spectrometry analysis of semi-pure mixtures of **1**–**3** and **5**–**10**.

**Microcystin**	**Measured *m/z***	**Molecular Formula**	**Calculated *m/z***	**Deviation (ppm)**
MC-WR (**1**)	1068.5465	C54H74N11O12	1068.5513	−4.5
MC-WA (**2**)	1005.4650	C51H66N8O12Na	1005.4692	−4.3
MC-WAba (**3**)	1019.4836	C52H68N8O12Na	1019.4849	−1.3
MC-KynR (**5**)	1072.5431	C53H74N11O13	1072.5462	−2.9
MC-OiaR (**6**)	1084.5449	C54H74N11O13	1084.5462	−1.2
MC-NfkR (**7**)	1100.5449	C54H74N11O14	1100.5411	+3.4
MC-KynA (**8**)	1009.4670	C50H66N8O13Na	1009.4642	+2.8
MC-OiaA (**9**)	1021.4634	C51H66N8O13Na	1021.4642	−0.8
MC-NfkA (**10**)	1037.4598	C51H66N8O14Na	1037.4591	+0.8

Figure S5. Downfield region of the 1H NMR spectrum of MC-NfkA (**10**; 600 MHz; CD3OH; electronic sculpturing suppression of the O*H*/*H*2O solvent peak and continuous wave suppression of the C*H*D2OH solvent peak).

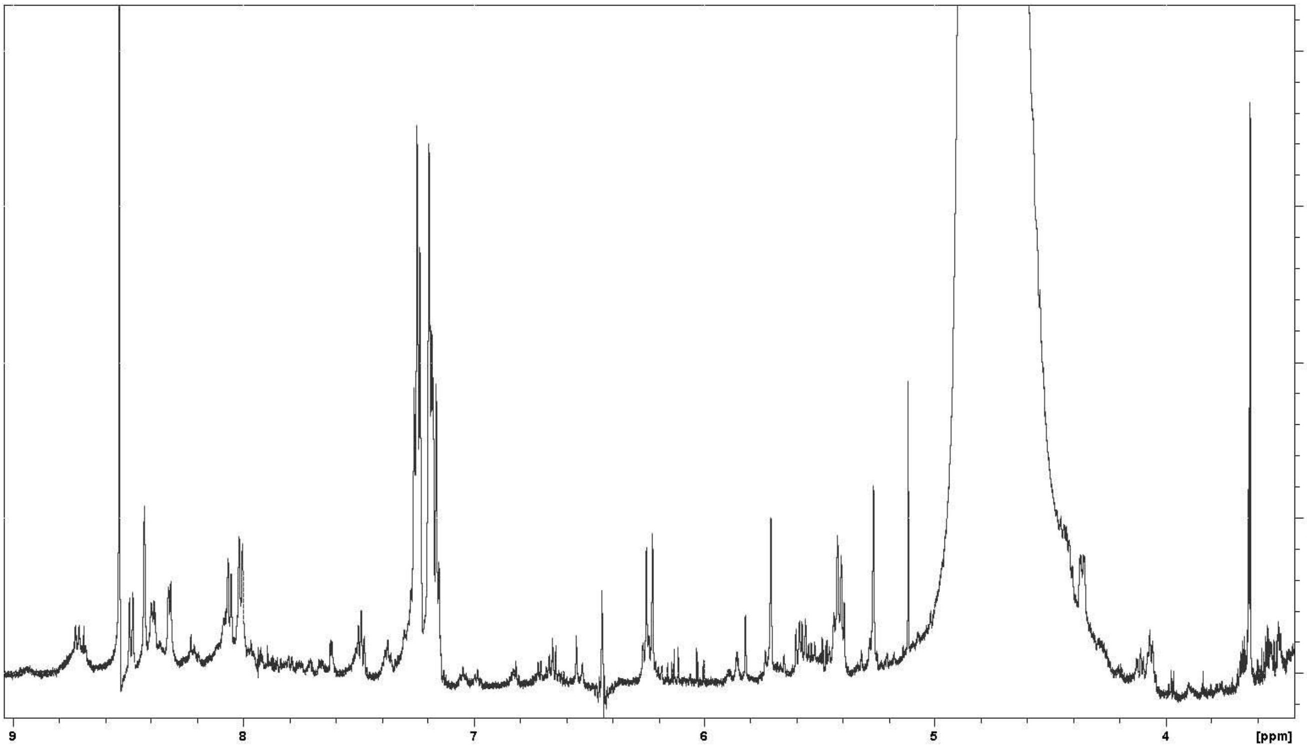

Figure S6. Upfield region of the ^{1}H NMR spectrum of MC-NfkA (**10**; 600 MHz; CD3OH; electronic sculpturing suppression of the O*H*/*H*2O solvent peak and continuous wave suppression of the C*H*D2OH solvent peak).

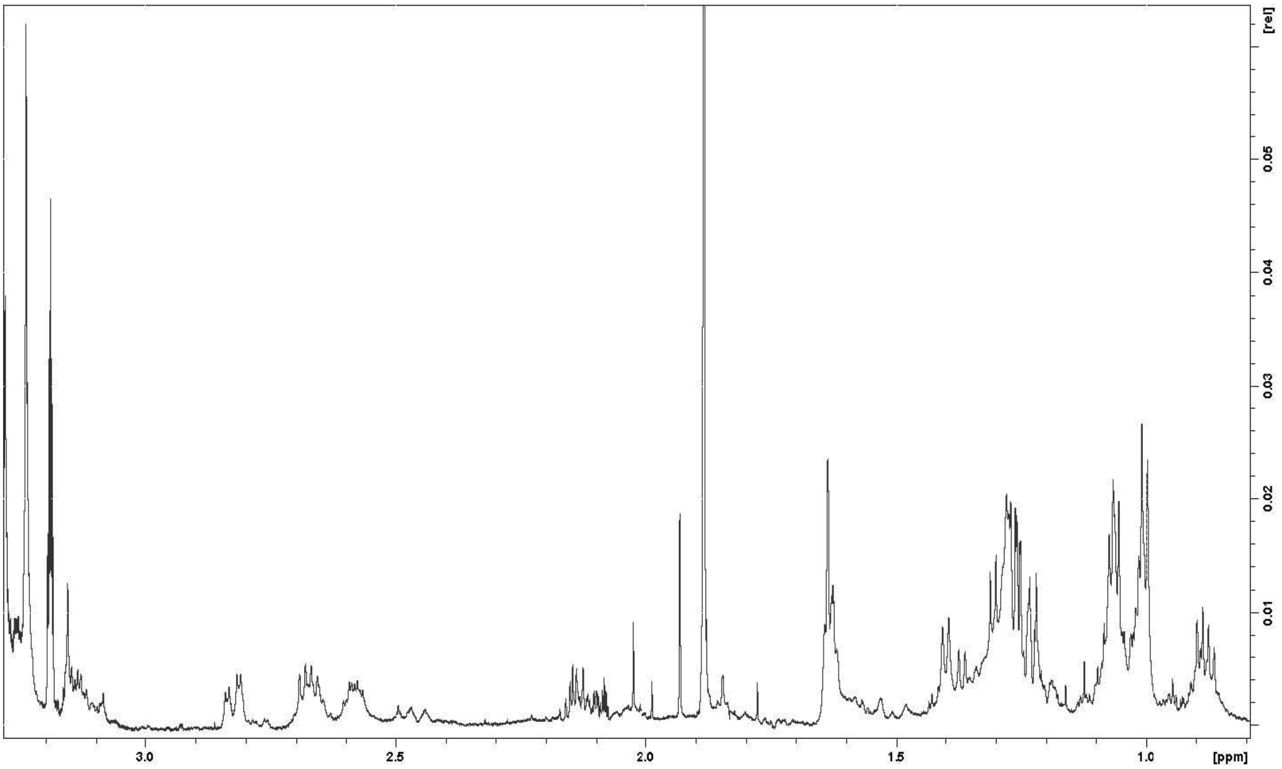

Figure S7. Downfield region of the ^{1}H-^{13}C HSQC NMR spectrum of MC-NfkA (**10**; CD3OH; continuous wave suppression of the O*H*/*H*2O solvent peak).

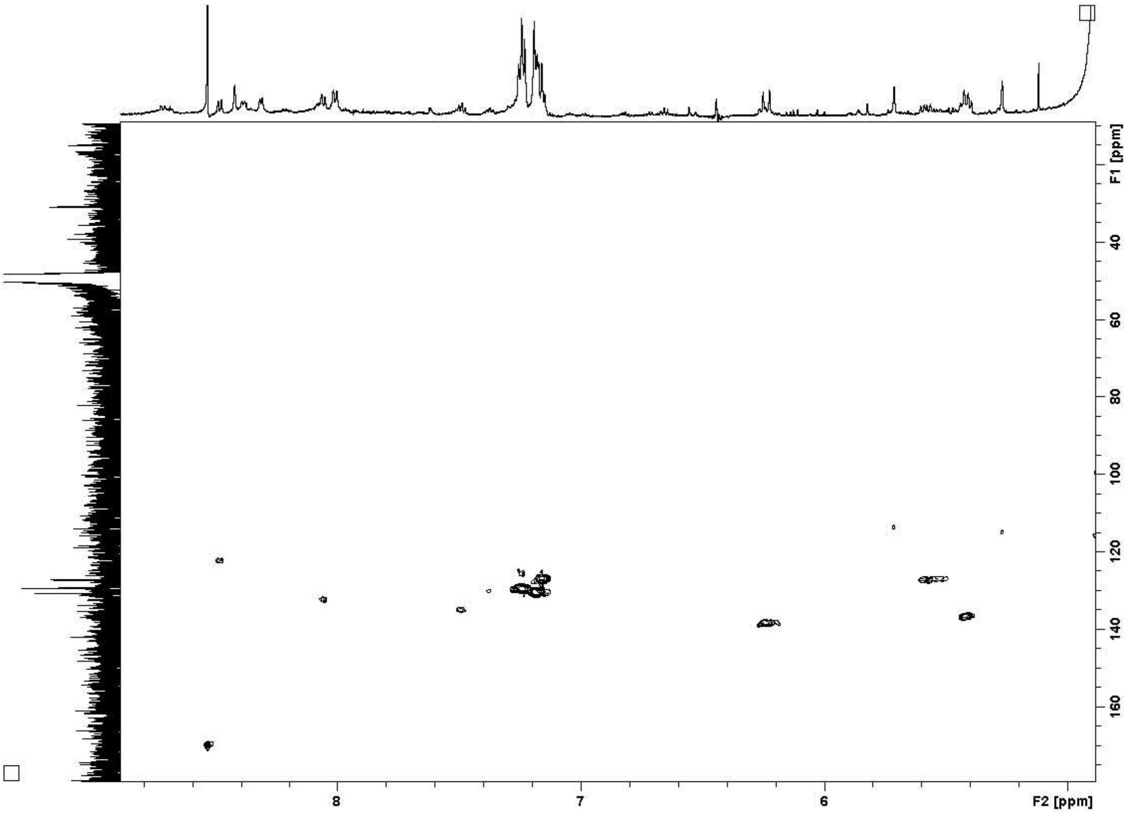

Figure S8. Upfield region of the ^{1}H-^{13}C HSQC NMR spectrum of MC-NfkA (**10**; CD_3OH; continuous wave suppression of the O*H*/*H*$_2$O solvent peak).

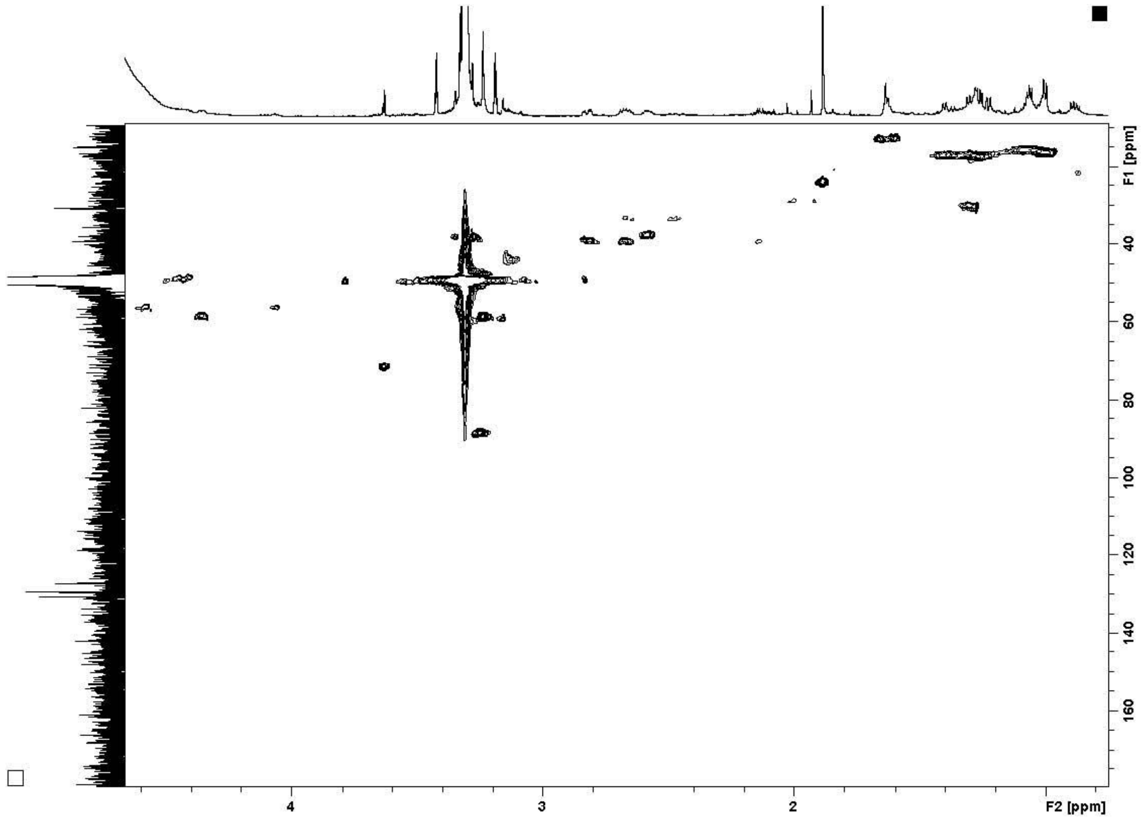

Figure S9. ^{1}H-^{13}C HMBC NMR spectrum of MC-NfkA (**10**; CD3OH).

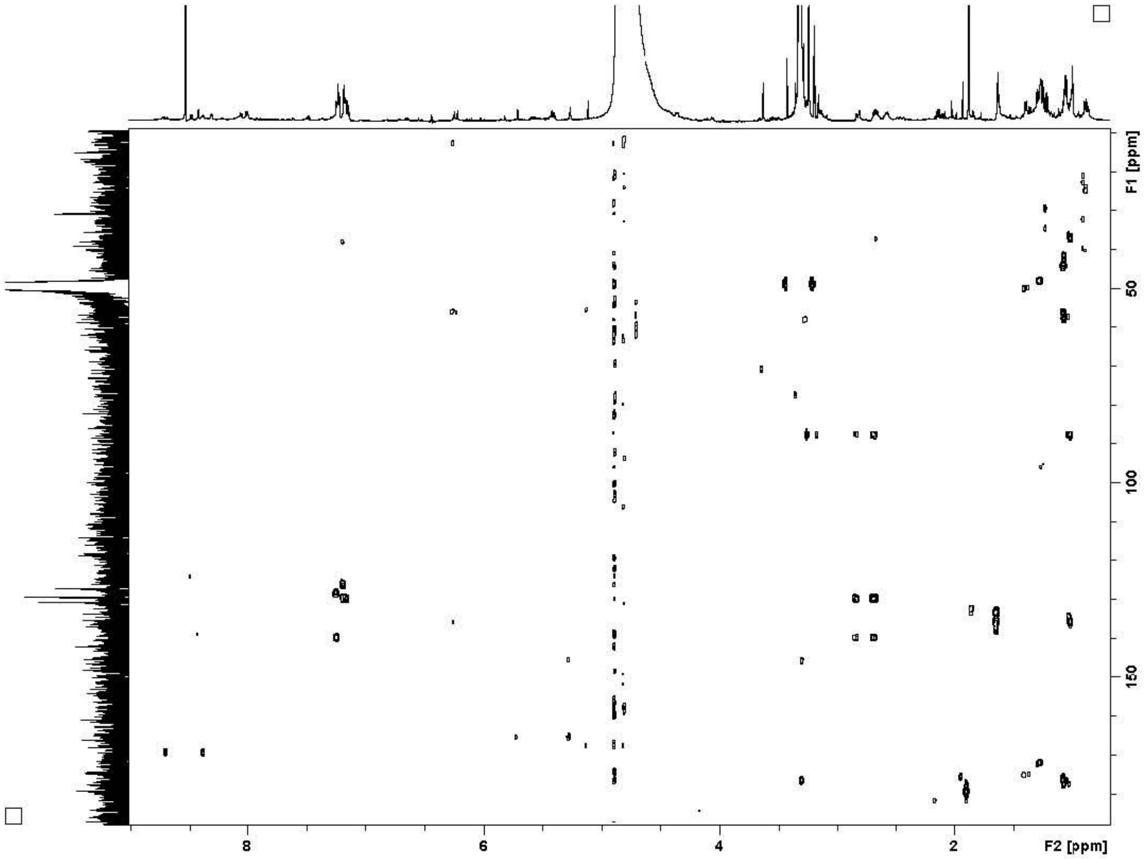

Figure S10. ^{1}H-^{1}H COSY NMR spectrum of MC-NfkA (**10**; CD3OH; continuous wave suppression of the O*H*/*H*2O solvent peak).

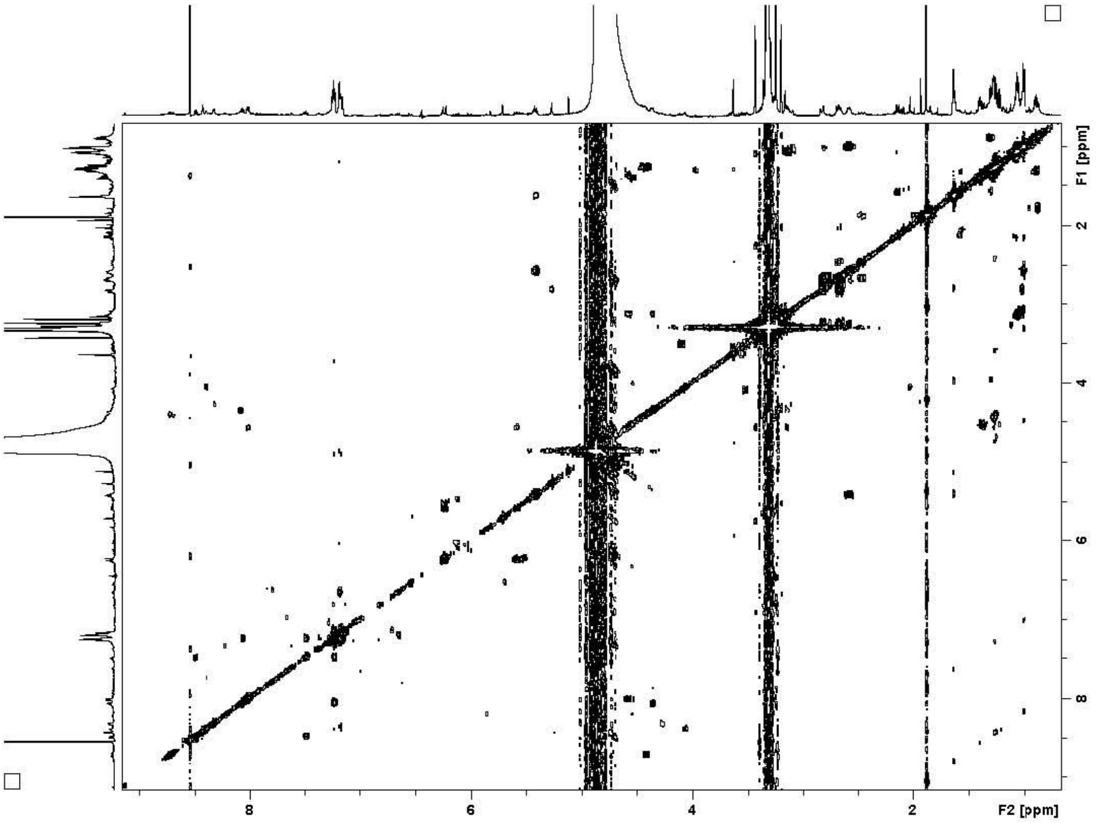

Figure S11. ^{1}H-^{1}H ROESY NMR spectrum of MC-NfkA (**10**; CD3OH; continuous wave suppression of the O*H*/*H*2O solvent peak).

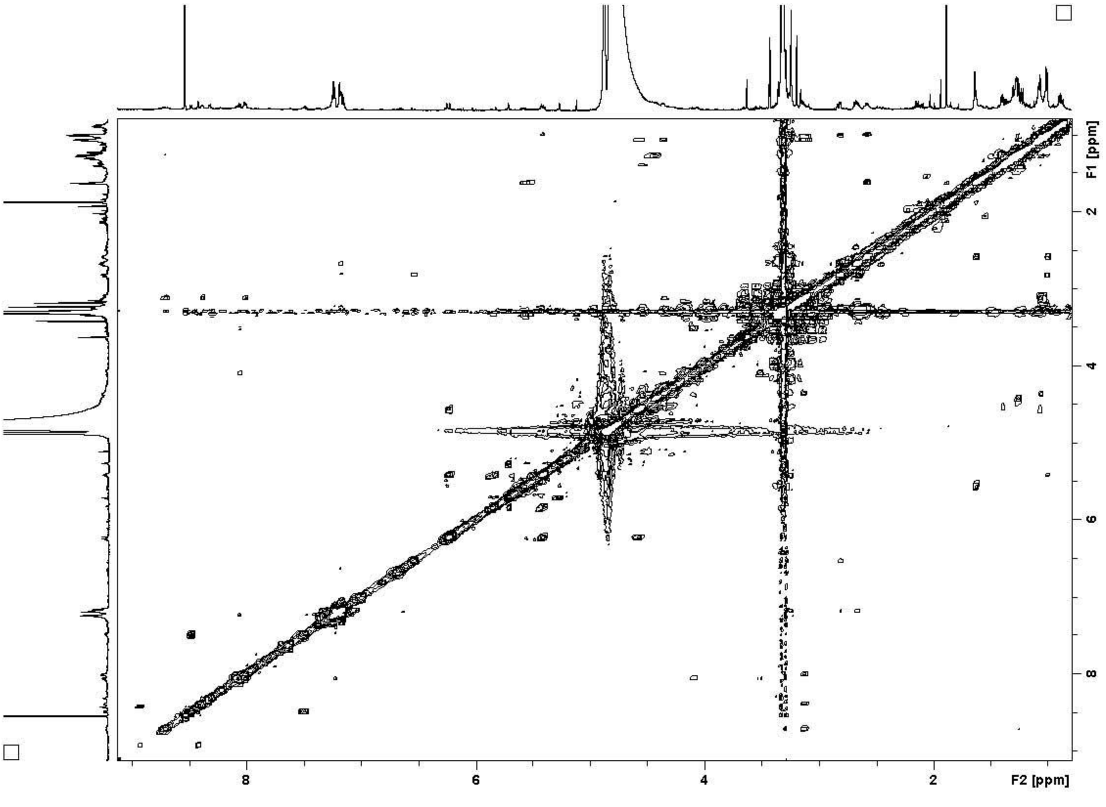

Figure S12. Graphs depicting the oxidation of MC-WA into MC-KynA, MC-OiaA and MC-NfkA through (**a**) diffusion with atmospheric oxygen, (**b**) the application of stirring and (**c**) the application of hydrogen peroxide.

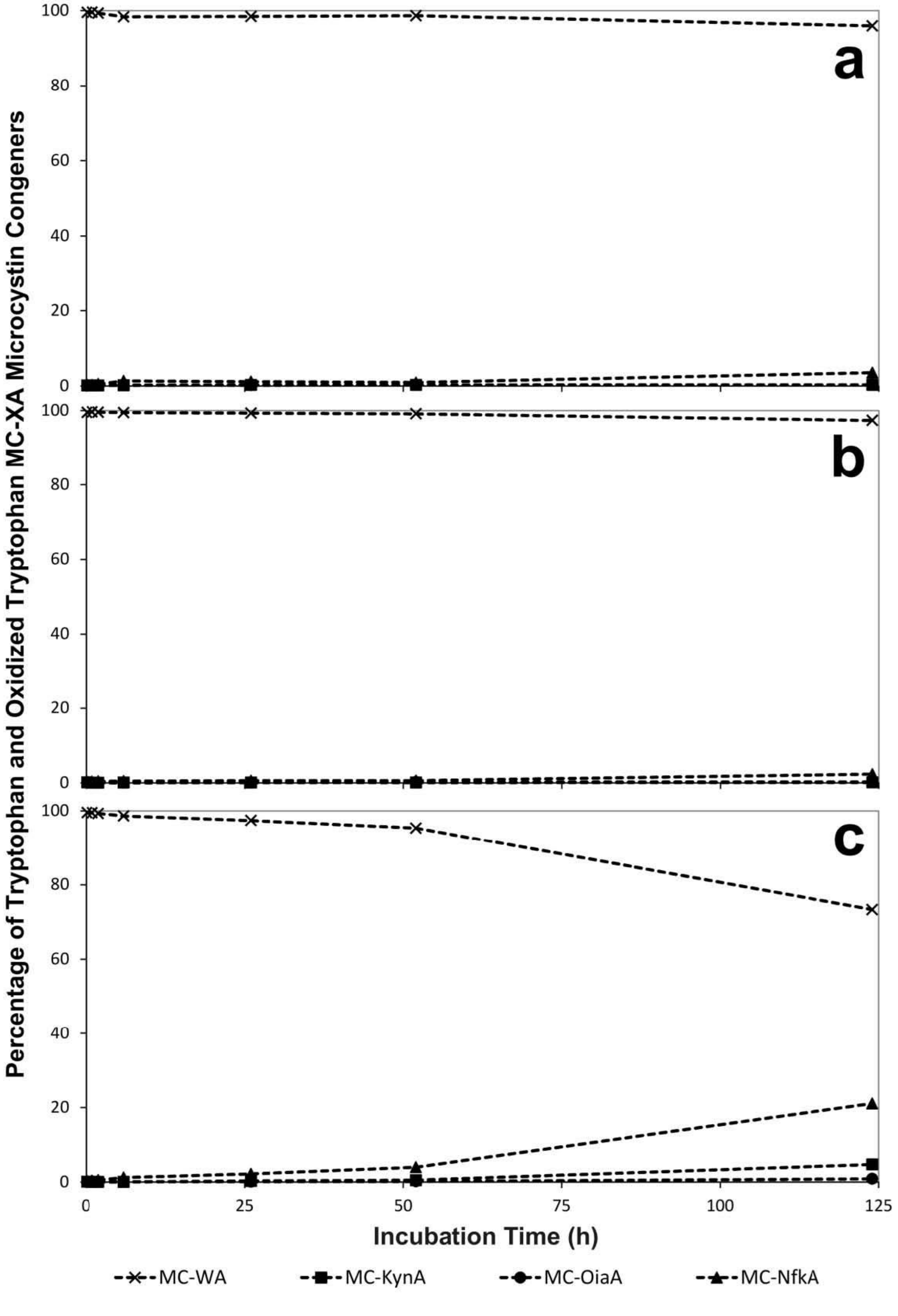

Supplement: Supplementary File 1 — Supplementary Materials (PDF, 746 KB) [file marinedrugs-11-03025-s001.pdf]
